# An Improved LC–MS/MS Method for the Analysis of Thirteen Cytostatics on Workplace Surfaces

**DOI:** 10.3390/ph14080754

**Published:** 2021-07-31

**Authors:** Maria Francisca Portilha-Cunha, Sara Ramos, Adrián M. T. Silva, Pedro Norton, Arminda Alves, Mónica S. F. Santos

**Affiliations:** 1 LEPABE—Laboratory for Process Engineering, Environment, Biotechnology and Energy, Faculty of Engineering, University of Porto, 4200-465 Porto, Portugal; mfcunha@fe.up.pt (M.F.P.-C.); aalves@fe.up.pt (A.A.); 2 Instituto de Saúde Pública, Universidade do Porto, Rua das Taipas 135, 4050-600 Porto, Portugal; saramos.89@gmail.com; 3 Laboratory of Separation and Reaction Engineering—Laboratory of Catalysis and Materials (LSRE–LCM), Faculty of Engineering, University of Porto, 4200-465 Porto, Portugal; adrian@fe.up.pt; 4 Departamento de Saúde Ocupacional, Centro Hospitalar Universitário São João, 4200-319 Porto, Portugal; pnorton@med.up.pt; 5 EPIUnit, Instituto de Saúde Pública, Universidade do Porto, Rua das Taipas 135, 4050-600 Porto, Portugal; 6 Departamento de Ciências da Saúde Pública e Forenses e Educação Médica, Faculdade de Medicina, Universidade do Porto, 4200-319 Porto, Portugal

**Keywords:** antineoplastic drugs, cytotoxic drugs, occupational exposure, healthcare workers, surface contamination, environmental contamination, analytical method, wipe sampling

## Abstract

Cytostatics are drugs used in cancer treatment, which pose serious risks to healthcare workers. Dermal absorption via surface contamination is the key exposure route; thus, rapid, reliable, and validated analytical methods for multicomponent detection are crucial to identify the exposure risk. A surface-wipe-sampling technique compatible with hospitals’ safety requirements (gauze, 1 mL isopropanol) and a fast and simple extraction method (1 mL acetonitrile, 20 min ultrasonic bath, evaporation, reconstitution in 200 µL acetonitrile), coupled with liquid chromatography–tandem mass spectrometry analysis, were developed. It allowed identification and quantification of 13 cytostatics on surfaces: cyclophosphamide, doxorubicin, etoposide, ifosfamide, paclitaxel, bicalutamide, capecitabine, cyproterone, flutamide, imatinib, megestrol, mycophenolate mofetil, prednisone. Good linearity, sensitivity, and precision were achieved (R^2^ > 0.997, IDLs < 4.0 pg/cm^2^, average CV 16%, respectively). Accuracy for four model surfaces (melamine-coated wood, phenolic compact, steel 304, steel 316) was acceptable (80 ± 12%), except for capecitabine and doxorubicin. Global uncertainty is below 35% for concentrations above 100 pg/cm^2^ (except for capecitabine and doxorubicin)—a guidance value for relevant contamination. Method application in a Portuguese university hospital (28 samples) identified the presence of seven cytostatics, at concentrations below 100 pg/cm^2^, except for three samples. The widespread presence of cyclophosphamide evinces the necessity to review implemented procedures.

## 1. Introduction

Nowadays, the administration of cytostatics, also referred to as cytotoxic and antineoplastic drugs, is essential in cancer treatment due to the inhibition of neoplasia evolution. Nevertheless, they are relatively nonspecific, affecting simultaneously malignant and normal cells, which may lead to adverse health effects on both treated patients and exposed health professionals. In fact, these drugs are classified as potentially carcinogenic, mutagenic, and/or teratogenic to humans [1]. Besides short-term effects, low-dose exposure might be dangerous if prolonged and constant [2]. Nurses, responsible for administering the medication, and pharmacy professionals, responsible for its preparation, are particularly exposed. However, other workers connected to the cytostatic circuit (manufacturing, transport, storage, preparation, administration, waste disposal, sanitation), normally less protected, may also be exposed [3,4]. Furthermore, the International Agency for Research on Cancer (IARC) estimates that an increase of approximately 50% in new cases is projected to occur between 2020 and 2040, in relation to demographic changes alone [5]. Therefore, the expected rise in the prescription of cytostatics and in professional’s workload will continue to increase the risk posed by exposure to these drugs.

Studies worldwide describe cytostatic contamination in a wide range of external surfaces, from laminar flow cabinets (where they are prepared) to administration areas, despite the use of personal protective equipment, ventilated engineering controls, and isolators [6,7,8,9]. In fact, dermal absorption by direct skin contact with contaminated surfaces and equipment is the primary way of exposure (ingestion due to contaminated hands and inhalation of aerosolized drugs can also happen) [6]. Despite the growing concern of European authorities for the past three decades, prevention is solely based on compliance with guidelines [10], since no exposure limit values have been defined for these drugs (yet, threshold guidance values set at the median value and at the 75th, 90th, or 99th percentile have been proposed [3,11,12]). Moreover, the pattern of cytostatic administration varies among countries, and most do not have in-depth research on this topic; for example, only two studies have been performed in Portuguese pharmacies and day-care hospitals [13,14]. Since the physicochemical characteristics of each drug impact their sorption on surfaces or air dispersion, a personalized study is essential. Hence, prevention programs based on periodic environmental monitoring of surface contamination by cytostatics are crucial to identify the exposure risk, implement preventive measures and validate decontamination practices (as required by Directive 2004/37/EC).

To ensure an adequate monitoring program, rapid, reliable, and validated analytical methods are needed. Important validation parameters include linearity, precision, accuracy, specificity, sensitivity, and uncertainty. Nevertheless, this is one of the major gaps in this research area since few methods described in the literature present data validation, and their comparison is challenging due to the lack of global uncertainty values associated with the results [15]. Consequently, limited accurate quantification and exposure risk assessment have been achieved.

Additionally, simultaneous detection of several cytostatics is increasingly important because their individual impacts on health differ considerably [16,17]. In this study, included cytostatics were bicalutamide (BIC), capecitabine (CAP), cyclophosphamide (CYC), doxorubicin (DOX), etoposide (ETO), flutamide (FLU), ifosfamide (IFO), imatinib (IMA), megestrol (MEG), mycophenolate mofetil (MMF), and paclitaxel (PAC). Although prednisone (PRE) and cyproterone (CYPR) do not belong to class L of the Anatomical Therapeutic Chemical code (antineoplastic and immunomodulating agents) [18], these drugs are commonly administered in combination with antineoplastics in cancer treatment, and hence, they were included in the method developed. CYC and ETO are classified as carcinogenic to humans (group 1) by the IARC; DOX is probably carcinogenic to humans (group 2A), while IFO and PRE are not classifiable as to its carcinogenicity to humans (group 3). The remaining drugs are not yet classified due to the lack of toxicological information.

There is also a need for lower detection limits and good extraction techniques since the principle of “as low as reasonably achievable” should be followed to avoid potential effects. Wipe sampling is generally employed since it is very useful to recover residual contaminants from surfaces, despite the variety of materials or type and volume of desorbing solution used. LC has been the preferred instrumental technique due to its sensibility for simultaneous cytostatic detection, allowing lower detection limits than available alternatives [15]. Its fast quantification and the low or null volatility of cytostatics are also relevant. In fact, few studies utilize GC since it requires the derivatization of the cytostatics (turning them into volatile compounds). The inductively coupled plasma method is only employed for the quantification of cytostatics with platinum. As a confirmatory technique, MS has been the preferred detector since it provides the necessary structural information to complement the separation by LC.

The present study aimed at developing an analytical methodology, comprising a wipe sampling procedure compatible with hospitals’ safety requirements, which would allow the simultaneous identification and quantification of 13 cytostatics on workplace surfaces of the cytostatic circuit. By assessing the method’s accuracy (by calculating the percent recovery of the compounds from different surfaces) and estimating its global uncertainty, this study contributes to filling several gaps in this research area, clearly adding to its value. Moreover, a search in the SCOPUS database for analytical methods developed for the analysis of cytostatics on workplace surfaces in the last five years revealed that no methods have been described for eight of the considered compounds: BIC, CAP, CYPR, FLU, IMA, MEG, MMF, and PRE. This lack of information further highlights the novelty of the present work. Additionally, a preliminary evaluation of the presence of the target cytostatics in a university hospital from northern Portugal was conducted.

## 2. Results and Discussion

### 2.1. Validation Parameters of the Chromatographic Method

The chromatographic and MS data, and some validation parameters concerning the analysis of the 13 cytostatics by LC–MS/MS are compiled in Table 1. Good linearity (R^2^ > 0.997; 0.2–200 ng) was generally attained for all compounds using the internal standard calibration approach. Displayed IDLs varied between compounds, but all were in the order of pg/cm^2^. These were obtained by dividing mass values by the standard sampling area (100 cm^2^) to be comparable with concentration values (mass per sampled area) of real contamination. The lowest IDLs were achieved for BIC and MMF (0.1 pg/cm^2^), while the highest were recorded for CYPR and IMA (4.0 and 2.0 pg/cm^2^, respectively). No IDL values were found in the literature for BIC, CAP, CYPR, FLU, IMA, MEG, MMF, and PRE on workplace surfaces. Apart from ETO, the remaining cytostatics are some of the most studied in this field; thus, various values were found. When employing LC–MS/MS, lower IDLs were found for CYC (c.a. 0.3 pg/cm^2^ versus 1.7 pg/cm^2^ in the present study) [16,19,20,21,22,23,24], while the only value for ETO (1.3 pg/cm^2^) was higher than the one obtained in the present work (0.4 pg/cm^2^) [23]. Regarding DOX, IFO, and PAC, although often slightly higher, the achieved values (0.9, 1.6, and 0.4 pg/cm^2^, respectively) are within those reported in the literature [16,19,20,21,22,23,24]. Additionally, only a study employing HPLC-UV was retrieved [25], which generated substantially higher IDLs (20–100 pg/cm^2^ for CYC, IFO, and PAC) than any of the studies with LC–MS/MS, reinforcing its lower method sensitivity. Since all 13 values obtained through the present method are in the same order of magnitude, it can be stated that good method sensitivity was achieved for all analyzed cytostatics.

### 2.2. Optimization of the Extraction Methodology

Initially, extraction conditions were only investigated for CYC, DOX, ETO, and PAC, due to their frequent use in current chemotherapy preparations by the hospital unit involved in the study, as well as worldwide; their previous study in the literature; their IARC classification (CYC and ETO are carcinogenic to humans, while DOX is probably carcinogenic to humans). Regarding the extraction solvent, although isopropanol, ethyl acetate, and nitric, formic or hydrochloric acid have been utilized, the most common are water, methanol and ACN [15]. Since water would not allow the concentration of the sample by solvent evaporation, and methanol promotes PAC hydrolysis, these solvents were ruled out. Therefore, different volumes of ACN were tested, and 1 mL was chosen as increasing the volume did not affect recoveries (preliminary data, not shown).

**Figure 1:**
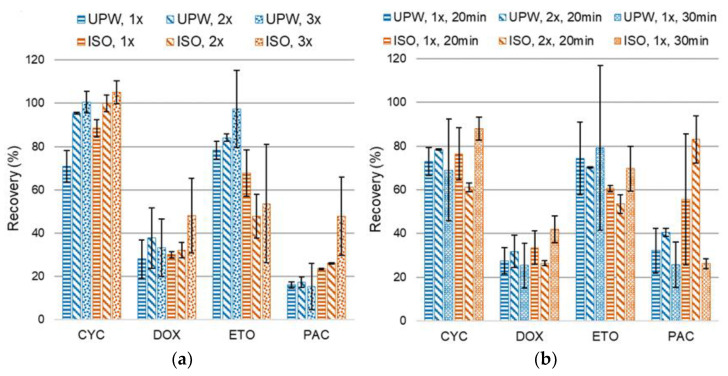
Optimization of the extraction efficiency of cyclophosphamide (CYC), doxorubicin (DOX), etoposide (ETO), and paclitaxel (PAC) from the gauze: (**a**) variation of number of extraction steps using vortex, for ultrapure water (UPW) and isopropanol (ISO); (**b**) variation of number and time of extraction steps using ultrasonic bath, for UPW and ISO. Reference protocol: one gauze embedded with 1 mL UPW or ISO spiked with 40 µL of 500 ng/mL of all target cytostatics, 1 mL of extraction solvent (acetonitrile), different extraction technique, evaporation, and reconstitution. Bars represent standard deviation.

For both extraction techniques assessed (vortex and ultrasonic bath), the performance of more than one step was considered to evaluate the possibility of increased recoveries. That was somewhat anticipated since each additional step involves further addition of extraction solvent and increases global extraction time. Considering that the nature of the desorbing solution (UPW or ISO) could also impact the extraction of cytostatics from the gauze using acetonitrile as extraction solvent, all experiments were carried out for both desorbing solutions as portrayed in Figure 1. With regard to tests with a vortex (Figure 1a), employing more than one extraction step (3 min each) did not change recoveries significantly. Nevertheless, CYC was noticeably more recovered in the presence of both water and isopropanol, and PAC was a little more recovered in the presence of isopropanol. Furthermore, only PAC in the presence of isopropanol seemed to be more recovered with three steps, although not by much, considering the standard deviation of triplicate tests. Hence, only one step seems adequate as it would not be substantially advantageous to consume more solvent and make the extraction process longer. Similarly, employing more steps of the ultrasonic bath (20 min each) did not provide considerable changes in recovery efficiency either (Figure 1b). Therefore, the same rationale was applied to choose one step as adequate.

The final choice of the agitation technique was based on the comparison of recoveries for one step of each technique (horizontal-dashed bars in Figure 1). It can be observed that PAC was considerably more recovered by ultrasonic bath for both desorbing solutions, and no significant differences were observed for the other cytostatics. Although ultrasonic bath takes more time than vortexing for individual samples, it allows simultaneous processing of larger numbers of samples and, consequently, reduces overall extraction time. On the other hand, vortexing is laborious and cannot be automated. Thus, the ultrasonic agitation technique was chosen to carry subsequent tests.

Regarding the duration of the ultrasonic bath, preliminary tests indicated that 10 min yielded lower recoveries (data not shown); thus, 20 min was selected as the minimum time. When ultrasonic bath duration was increased to 30 min, CYC, DOX, and ETO were slightly better recovered, but PAC was not, for which a reduction of c.a. 50% was attained in isopropanol (Figure 1b). Since lengthier processing times are not beneficial and energetic costs associated with ultrasonic agitation can be expensive, longer durations were not further assessed and 20 min was deemed acceptable.

The final conditions to extract the cytostatics from the gauze were 1 mL ACN, as extraction solvent, and one step of 20 min of the ultrasonic bath as agitation technique.

### 2.3. Optimization of the Desorption Methodology

**Figure A1:**
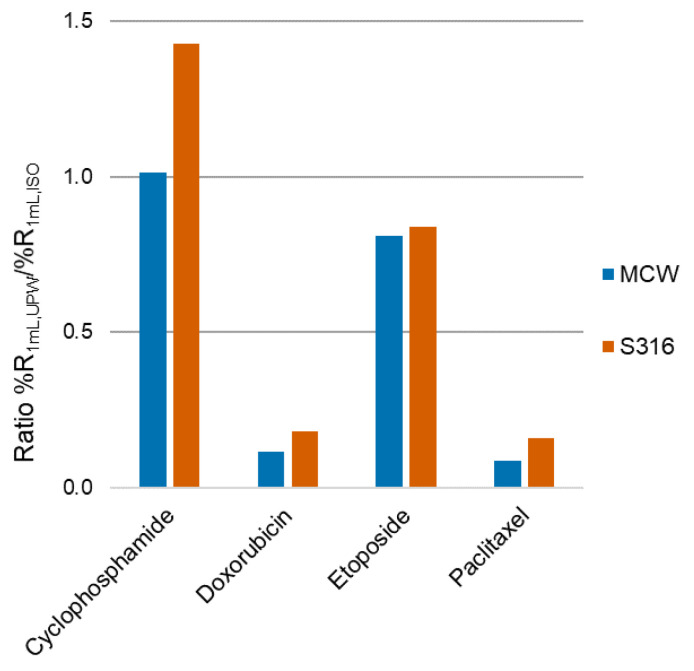
Ratio between mean recoveries of tests using 1 mL ultrapure water (UPW) and tests using 1 mL isopropanol (ISO), from two regular surfaces: melamine-coated wood (MCW) and steel 316 (S316). Reference protocol: 100 cm^2^ of surface spiked with 40 µL of 500 ng·mL^−1^ of all target cytostatics, wipe sampling with one gauze embedded with 1 mL ISO or 1 mL UPW, 1 mL of extraction solvent (acetonitrile), ultrasonic bath for 20 min, evaporation, and reconstitution.

**Figure A2:**
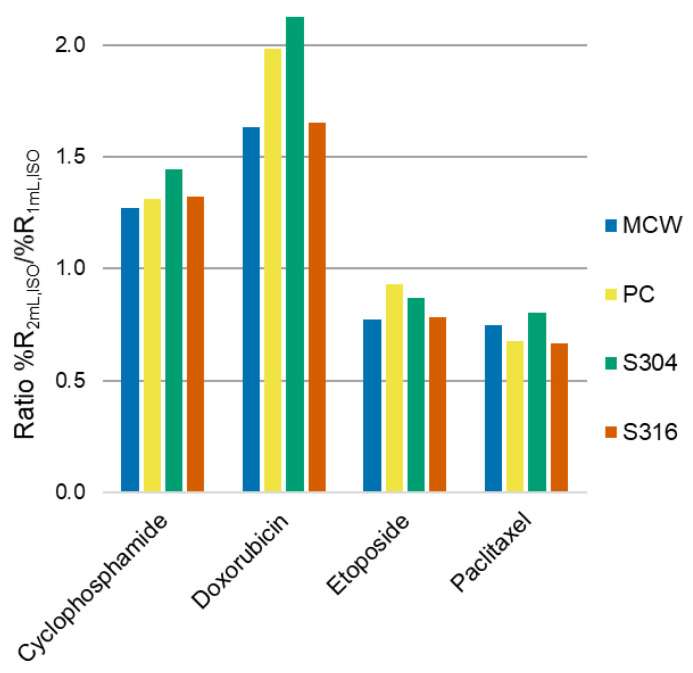
Ratio between mean recoveries of tests using 2 mL isopropanol (ISO) and tests using 1 mL ISO, from four regular surfaces: melamine-coated wood (MCW), phenolic compact (PC), steel 304 (S304), and steel 316 (S316). Reference protocol: 100 cm^2^ of surface spiked with 40 µL of 500 ng·mL^−1^ of all target cytostatics, wipe sampling with one gauze embedded with 2 mL ISO or 1 mL ISO, 1 mL of extraction solvent (acetonitrile), ultrasonic bath for 20 min, evaporation and reconstitution.

The wipe sampling procedure, similar to most studies, consisted of cleaning a predefined area, in different directions, with a wetted adsorbent material, since a dry sampling medium would have little tendency to extract cytostatics from surfaces. Common materials for this purpose include gauze/wipes/tissues, cellulose filter papers, and swabs [15]. The decision to utilize gauze was based on their low cost and availability in hospitals. Regarding the surface desorbing solution, isopropanol, methanol, water, and ACN are the most common [15]. Water, ethanol, and isopropanol were considered because they are polar solvents (a fundamental requirement for the extraction of polar compounds such as cytostatics), as well as due to their low cost and availability in hospitals, and the fact that they do not pose increased risks for workers or patients. However, preliminary results revealed that ethanol was not able to recover PAC (data not shown), which was likely hydrolyzed, similarly to what happens in the presence of methanol. Consequently, it was excluded from further tests. Isopropanol proved to be advantageous as it recovered significantly more DOX and PAC from two model surfaces (MCW and S316), while ETO was similarly recovered, and CYC was either equally or slightly more recovered with water (Figure A1, Appendix B). Regarding volume, using 2 mL isopropanol increased DOX recoveries by at least 1.5 fold, whereas differences for other cytostatics were limited—ETO and PAC were negatively affected (maximum 0.3 fold), while CYC was positively affected (maximum 0.5 fold) (Figure A2, Appendix B). Nevertheless, DOX recoveries from surfaces remained very low in comparison with the other 12 cytostatics, which were satisfactorily recovered with 1 mL and under the conditions previously described. Additionally, increasing the volume entails more costs and would increase evaporation time, which already is the longest step in the processing time. From a broad perspective, it does not pay off to double isopropanol volume; thus, 1 mL was selected.

Accuracy values compiled in Table 1 reflect both the effect of the extraction process and of the matrix on the recovery of cytostatics from the four surfaces studied. Of the 13 studied compounds, ETO, CYC, BIC, CYPR, FLU, IFO, MEG, MMF, and PRE have high values (greater than 70%), while IMA and PAC have satisfactory values (54% and 68%, respectively). In contrast, care should be taken in the interpretation of DOX and CAP analysis: while DOX was not efficiently recovered (24%), CAP has shown probable matrix effects or solvent interference reaching percent recoveries significantly higher than 100%. As before, no percent recoveries were found in the literature for BIC, CAP, CYPR, FLU, IMA, MEG, MMF, and PRE. Additionally, most studies report only recoveries acquired from the sampling medium [16,19,22,25,26,27], and others only provide the mean recovery of all studied cytostatics [17,23,24]. Regarding recoveries from surfaces [21,28,29,30], no values were found for ETO, while values ranged from 50 to 103% for CYC, 26–61% for DOX, 31–100% for IFO, and 16–109% for PAC (when sampling from plates of stainless steel, Formica^®^, and vinyl in different conditions). The value achieved in the present study for CYC (87%) is similar to most published recoveries, while values for DOX, IFO, and PAC (24%, 82%, and 68%, respectively) are somewhat lower than most reported values, although being within the aforementioned ranges. It is relevant to point out that, similar to this study, recoveries for DOX reported in the literature are significantly lower than values for other cytostatics simultaneously analyzed. However, no attempts have been made to clarify such discrepancies.

**Figure A3:**
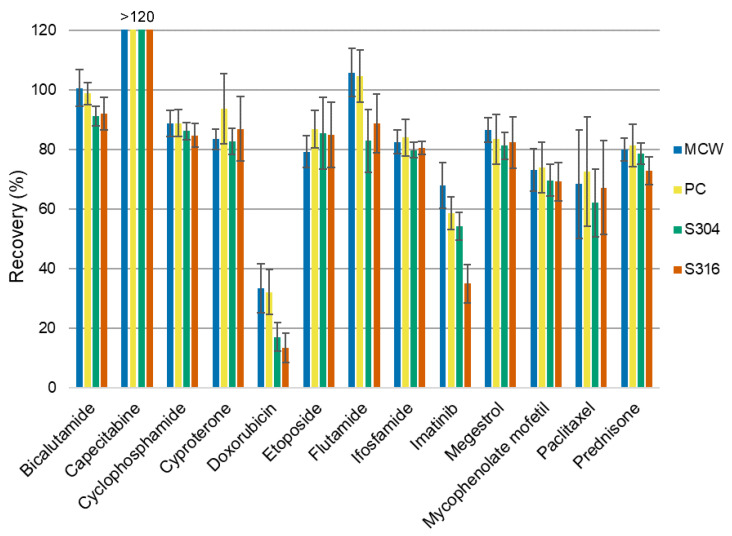
Mean recoveries for 13 cytostatics extracted from four regular surfaces: melamine-coated wood (MCW), phenolic compact (PC), steel 304 (S304), and steel 316 (S316). Extraction protocol: 100 cm^2^ of surface spiked with 40 µL of 500 ng·mL^−1^ of all target cytostatics, wipe sampling with one gauze embedded with 1 mL isopropanol, 1 mL of extraction solvent (acetonitrile), ultrasonic bath for 20 min, evaporation and reconstitution. Bars represent standard deviation.

In fact, it should be acknowledged that many factors impact surface desorption capacity, other than extraction and instrumental methodologies [15]. Issues related to the surface itself, such as material’s type and condition, are usually the main concern. In fact, some studies have reported that surface roughness plays a crucial role in the desorbing potential of wipe sampling for some drugs. Specifically, a decrease in recoveries has been observed for surfaces with greater roughness, which appears to be a consequence of worn surfaces [21,28]. Yet, large variations in recoveries within surfaces with similar roughness indicate that other parameters are also important. For example, the influence of surface material should be considered because it defines surface chemistry, which is particularly relevant for wipe sampling [24,30]. In the case of the four surfaces investigated, recoveries were similar for most cytostatics (Figure A3, Appendix B). However, values were noticeably higher for MCW and PC rather than for the steel surfaces (S304 and S316) for DOX, CAP, and FLU, while in the case of IMA, S316 showed lower recoveries than the other surfaces. Nevertheless, precision was considered acceptable, with an average coefficient of variation of 16% for all assays (minimum 5% for CYC, maximum 46% for DOX), considering different days and different surfaces. Additionally, a study concluded that personnel with more training and experience achieved significantly higher recoveries [28]. That highlights the importance of adequate training of personnel conducting sampling to attain more accurate results, as wiping is frequently performed by workplace employees.

### 2.4. Global Uncertainty Associated with the Results

**Figure 2:**
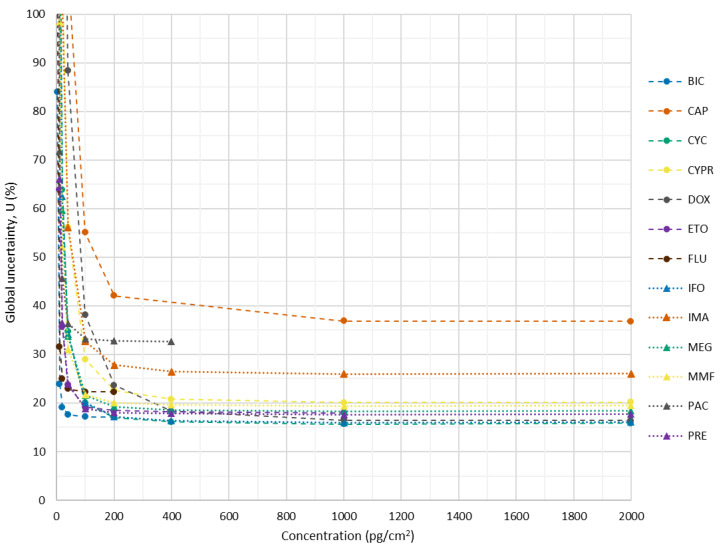
Global uncertainty of the analytical methodology for quantification of 13 cytostatics on surfaces by LC–MS/MS: bicalutamide (BIC); capecitabine (CAP); cyclophosphamide (CYC); cyproterone (CYPR); doxorubicin (DOX); etoposide (ETO); flutamide (FLU); ifosfamide (IFO); imatinib (IMA); megestrol (MEG); mycophenolate mofetil (MMF); paclitaxel (PAC); prednisone (PRE). Dashed lines are merely illustrative of the data trend.

**Figure A4:**
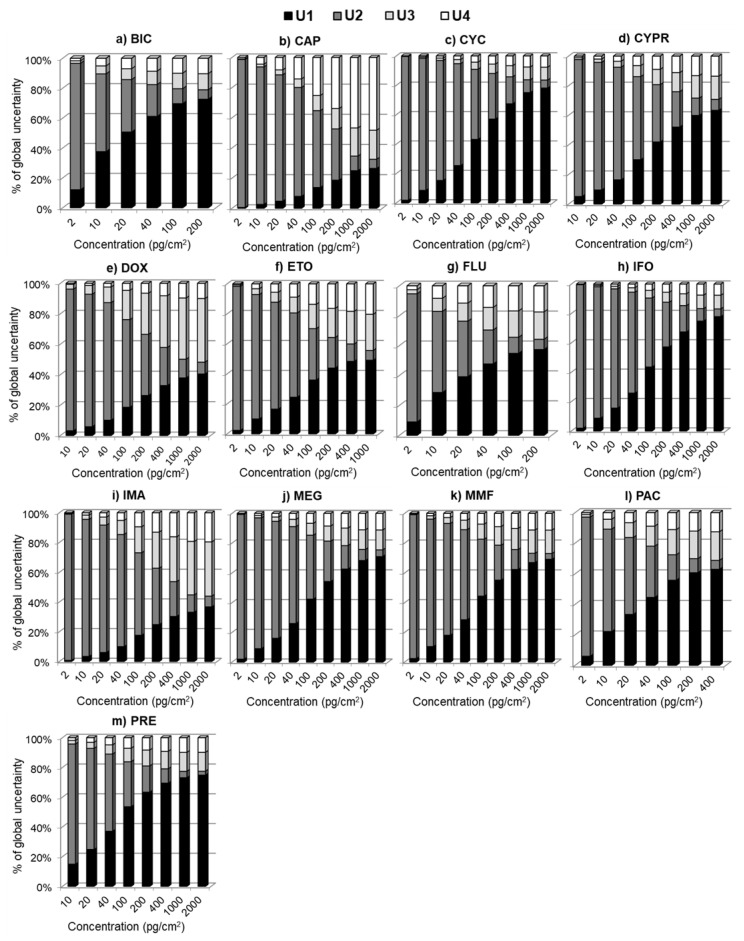
Relative weight of each individual source of uncertainty (bottom-up approach/EURACHEM) for quantification of 13 cytostatics in surfaces by LC–MS/MS: (**a**) bicalutamide (BIC); (**b**) capecitabine (CAP); (**c**) cyclophosphamide (CYC); (**d**) cyproterone (CYPR); (**e**) doxorubicin (DOX); (**f**) etoposide (ETO); (**g**) flutamide (FLU); (**h**) ifosfamide (IFO); (**i**) imatinib (IMA); (**j**) megestrol (MEG); (**k**) mycophenolate mofetil (MMF); (**l**) paclitaxel (PAC); (**m**) prednisone (PRE). U1-uncertainty associated with standard preparation; U2-uncertainty associated with the calibration curve; U3-uncertainty associated with precision; U4-uncertainty associated with accuracy.

Global uncertainty associated with results is a figure of merit extremely valuable, particularly when results from different methods are compared or when a maximum legal limit is under consideration. Individual contributions of the four considered sources to the global uncertainty are depicted in Figure A4. In the analysis of the 13 cytostatics, three main difficulties arise in uncertainty measurement: (1) the dependence of the uncertainty with the concentration; (2) the vicinity of the method detection limit, where most of the samples fall; (3) the difficulty of performing spiking experiments. For the uncertainty associated with the results obtained, using the EURACHEM methodology described in Section 3.5.2. and in Appendix A, the first problem is clearly observed in Figure 2. For concentrations above 100 pg/cm^2^, the global uncertainty is below 35%, except for CAP and DOX, and this result may be used by default. However, when concentrations are lower than 100 pg/cm^2^, global uncertainty increases, reaching about 200% for concentrations near 10 pg/cm^2^ for all cytostatics, except for DOX and CAP, which reach this value earlier (c.a. 20 pg/cm^2^).

One of the main topics of discussion among analysts has been whether to correct the results with the recovery obtained, and arguments in favor and against it are found in the literature. This is particularly important for CAP and DOX: %R is extremely high for the first (251 ± 81%) and extremely low for the second (24 ± 11%). In fact, using percent recoveries from spiking experiments may introduce errors related to difficulties of the spike solution to attain equilibrium, since it may not be so firmly bound to the matrix as the native analyte. Therefore, the surrogate recovery will tend to be higher than that of the native analyte. That circumstance would lead to a negative bias in a corrected analytical result. On the other hand, for low concentrations, a proportion of the analyte may be unrecoverable by virtue of irreversible adsorption on surfaces, leading to extremely low percent recoveries. In this study, considering the heterogeneity of the surfaces to be analyzed and the different physicochemical properties of the analytes, results are displayed in Table 2 without correction. However, percent recoveries (shown in Table 1) and global uncertainties (Figure 2) should be considered when interpreting the results.

### 2.5. Cytostatics Analysis of Workplace Surfaces

The validated analytical methodology was applied in the analysis of workplace surfaces as a preliminary assessment of healthcare workers’ risk due to occupational exposure to cytostatics in preparation and administration wards. For this application, no efforts were made to account for the impact of surface roughness or porosity nor surface chemistry, even though sampled locations were clearly distinctive (including working tables, an analytical balance, and a printer, for the preparation ward; trays, support carts, drug administrator monitors, and floor near beds/chairs of patients, for the administration unit).

As seen in Table 2, BIC, DOX, FLU, IMA, PAC, and PRE were not detected in any sample, and CAP, CYPR, ETO, and MMF were detected in three or fewer samples. The most detected cytostatic was CYC (16/28 samples), followed by MEG and IFO. In general, contamination appears to be higher in the preparation ward.

No exposure limit values have been defined for cytostatics; thus, in this study, 100 pg/cm^2^ was taken as an indicator to take corrective measures. This is supported by a safe reference value proposed in the literature (no positive biological samples were found for contamination levels below 100 pg/cm^2^, for CYC) [12]. and on a proposed substance-independent performance-based guideline (based on the 90th percentile of the contamination values) [3]. Thus, two CYC and one CYPR values were critical (the highest value detected was 174 pg/cm^2^). Additionally, the three samples where CYPR was detected were in the same area, near a patient’s chair, suggesting a spillage might have occurred recently. Even though results suggest contamination levels are generally quite low, a thorough discussion needs to be supported by an enlarged monitoring scheme. Nonetheless, the most relevant findings pertain to CYC, which is a carcinogenic cytostatic (IARC group 1) and whose presence alone evidences the necessity to take corrective measures.

These findings are in line with those reported by most studies performing cytostatic monitorization in the workplace [7], although with different cytostatics. In fact, a study in three Portuguese hospitals identified that the highest value (179 pg/cm^2^ for 5-fluorouracil (5-FU)) was in the laminar-flow hood, out of 40% contaminated samples (112 total) [13]. However, monitoring was limited because only 5-FU and platinum drugs (cis-, carbo- and oxaliplatin) were evaluated. On the other hand, a previous study in two Portuguese hospitals observed higher contamination in the administration unit [14]. Using CYC, PAC, and 5-FU as surrogate markers, at least one drug was quantified in 37% of the 327 samples, and more than one drug in 9%. Although not quantifiable, these markers were also detected in another 36% of samples, further supporting the importance of developing sensible analytical methods able to identify the frequently low values of cytostatics.

## 3. Materials and Methods

### 3.1. Chemicals and Reagents

BIC, CAP, CYC, CYPR, DOX, ETO, FLU, IFO, IMA, MEG, MMF, PAC, and PRE analytical standards of 98–99% purity were acquired from Sigma-Aldrich (St. Louis, MO, USA) and Cayman Chemical Company (Ann Arbor, MI, USA). Cyclophosphamide-d4 (CYC-d4) was used as an internal standard (IS) and supplied from Sigma-Aldrich (St. Louis, MO, USA). Acetonitrile (ACN), isopropanol, methanol, and Milli-Q water were of LC–MS grade and were supplied by Merck (Darmstadt, Germany). Formic acid was purchased from Sigma-Aldrich (St. Louis, MO, USA). Stock standard solutions were prepared at a concentration of 100 mg/L in ACN, due to the degradation of PAC in methanol. Working solutions were prepared at 10 mg/L in ACN. Commercial gauzes (10 × 20 cm, 70% viscose, and 30% polyester, 30 g/m^2^) were used for extraction assays.

### 3.2. Safety Considerations on Cytostatic Drugs Handling

For the preparation of standards, an exhaustive control on handling procedures, storage conditions, and safety rules was followed, as specified by the manufacturers. All the procedures were accomplished in a safety hood with vertical laminar airflow, and an absorbent paper was used to protect the work surfaces. All the materials in contact with the cytostatics were cleaned with isopropanol, and the dischargeable materials were treated as hazardous waste.

### 3.3. Extraction of Cytostatics from the Gauze

For the extraction of cytostatics from the gauze, three key conditions were investigated: agitation technique (vortex or ultrasonic bath); the number of extractions with ACN (1, 2, or 3 times); and time of ultrasonic bath (20 or 30 min). Experiments were performed for the two solvents added to moisten the gauze (water or isopropanol) in the surface desorption step, as it may also impact the extraction efficiency of cytostatics from the gauze. Accuracy was evaluated by spiking the gauze with 40 µL of a 500 ng/mL solution containing the target cytostatics. Best conditions were chosen based on recovery values given by Equation (1):

(1) $$\%R=\frac{Ms}{Mr}\times 100,$$

where *Ms* is the mass of cytostatic measured in the sample from a recovery test, and *Mr* is the mass of cytostatic measured in a standard, which was prepared with the same spike of the recovery test.

The final extraction procedure is briefly described by the following steps: (i) ¾ of one gauze was embedded in 1 mL of surface desorbing solution and it was placed in a 50 mL Falcon tube, as well as ¼ of dry gauze; (ii) then, 40 µL of 500 ng/mL of all target cytostatics were spiked and the extraction solvent was added (1 mL ACN); (iii) the content was shaken in an ultrasonic bath for 20 min; (iv) the organic solvent was recovered from the gauze and transferred to a 12 mL vial; (v) then, 20 µL of 1000 ng/mL IS were added to the extract (or to the combined extracts, in the case of more than one extraction steps); (vi) it was slowly evaporated to almost dryness under nitrogen gas; (vii) the remaining liquid was transferred to a 1.5 mL vial using ACN as washing solvent; (viii) the extract was evaporated to dryness; (ix) it was reconstituted in 200 μL ACN.

### 3.4. Desorption of Cytostatics from Contaminated Model Surfaces

For the desorption of cytostatics from surfaces, four different regular surfaces were assessed: melamine-coated wood (MCW), phenolic compact (made of phenolic resins and cellulosic fibers) (PC), steel 304 (S304), and steel 316 (S316). Only regular surfaces were used in desorption protocol development due to their easily determined area, compared to uneven surfaces, such as door handles or gloves.

In each experiment, 100 cm^2^ of the surface was spiked with 40 µL of a 500 ng/mL solution, containing the studied cytostatics. After solvent evaporation (c.a. 5 min), the cytostatics were desorbed from the surface by a technique known as wipe sampling. Each surface was wiped with ¾ of one gauze embedded in surface desorbing solution, using each ¼ to wipe in a different direction: horizontal, vertical, and diagonal. The remaining ¼ of dry gauze was used to pull the solvent that may have remained on the surface. All gauze parts were placed in a 50 mL Falcon tube for further treatment: extraction from the gauze was performed with the best conditions previously indicated in Section 3.3. For these experiments, 20 µL of 1000 ng/mL IS were spiked directly to the gauze after desorption from the surface to ensure that any possible loss due to the extraction procedure was accounted for.

Tests were performed under different surface desorbing protocols and the recoveries were determined according to Equation (1). The differences in the extraction protocols relied on the volume (1 or 2 mL) and type of desorbing solution (water or isopropanol). Water and an alcohol-based solvent were selected as desorbing solutions because they are already used in hospitals for microbial decontamination of surfaces. Acetonitrile was not tested since it is toxic in contact with skin and therefore is not compatible with hospitals’ safety requirements. In the end, 1 mL of isopropanol was selected to desorb the target cytostatics from the surfaces.

### 3.5. Instrumental Analysis

Analyses were carried out in liquid chromatography (Shimadzu Corporation; Tokyo, Japan) equipped with two Pumps LC-30AD, an Autosampler SIL-30 AC, an Oven CTO-20 AC, a Degasser DGU-20A5, a System Controller CBM-20A, an LC solution version 5.41SP1, and a triple quadrupole mass spectrometer detector Shimadzu LCMS-8040 (LC–MS/MS). Data were acquired and processed using the LabSolutions software package.

Separation was performed with a Luna C18 column (150 × 2.1 mm ID, particle size 5 μm; Phenomenex). The mobile phase composition consisted of a binary mixture of water (A) and methanol (B), both acidified with 0.1% formic acid. Gradient elution started at 5% B, increased to 20% B in 15 min, and then to 45% B in 15 min (30 min), reaching 100% in another 9 min (39 min). After 2 min at 100% B, the initial conditions were regained (4 min), and the system was stabilized for 5 min. The flow rate was set at 0.2 mL/min, and the injection volume was 5 μL.

An electrospray ionization source was operated in a negative (BIC and FLU) and positive (remaining cytostatics) modes. The precursor ions [M + H]^+^/[M − H]^−^, and the two most abundant fragments were used for identification (transition 2) and quantification (transition 1) of the target analytes (detailed information in Table 1). Optimized parameters were cone voltage (4.5 V for positive and −3.5 V for negatively ionized compounds), collision energy (from 10 to 50 eV), 3.0 dm^3^/min for nebulizing gas flow, 7.5 dm^3^/min for drying gas flow, 400 °C for block temperature, and 250 °C for desolvation line temperature.

#### 3.5.1. Validation Procedure: Quality Control/Quality Assurance

Calibration was performed over a mass range from 0.2 to 200 ng (equivalent to 2–2000 pg/cm^2^) using nine calibration points. Internal standard quantification was performed using CYC-d4 as a surrogate for all cytostatics.

The instrumental detection limits (IDLs) and the instrumental quantification limits (IQLs) were determined for a signal-to-noise ratio of 3 and 10, respectively, based on the analytical responses obtained for the standard of 20 ng of each cytostatic drug. Precision was estimated as the worst-case scenario, considering the coefficient of variation obtained from the recovery assays of 16 measurements on different days and with different samples. Solvent blanks did not contain any of the investigated analytes, indicating neither environment contamination nor carry-over effect during LC–MS/MS runs.

#### 3.5.2. Global Uncertainty

To estimate the global uncertainty associated with the quantification of cytostatics on surfaces by LC–MS/MS, the bottom-up approach proposed by the International Organization for Standardization and adopted by the EURACHEM-CITAC Guide was applied [31]. Four sources of uncertainty were considered: the uncertainty associated with the preparation of standards (estimated using the error propagation law for the different dilution steps from the stock standard solution); the uncertainty associated with the calibration curve (calculated for the different mass levels of the standards); the uncertainty associated with the precision of the method (estimated as the average result of the relative standard deviation of recovery assays); the uncertainty associated with the accuracy (calculated as the average percent recovery obtained within all the experiments). Detailed equations can be found in Appendix A.

### 3.6. Cytostatics Analysis of Workplace Surfaces

The method developed and validated was applied to 28 wipe samples from a university hospital in northern Portugal. The sampling points were chosen based on direct and passive observations of the daily practices of healthcare workers from the pharmacy and day-hospital units, aiming at the identification of the most potentially contaminated surfaces and/or more frequently handled or touched. Consequently, 12 different surfaces from the preparation ward and 16 from the administration unit were wiped. Sampling was performed on two weekdays.

## 4. Conclusions

A consistent wipe sampling technique (commercial gauze, 1 mL isopropanol) was successfully utilized for sampling workplace surfaces, avoiding the use of solvents with added toxicity to healthcare professionals. Likewise, the method developed for the extraction of cytostatics from the gauze (1 mL ACN, 20 min ultrasonic bath, evaporation, and reconstitution in 200 µL ACN) was fast, simple, and needed little volume of solvents. Overall, the extraction steps coupled with LC–MS/MS analysis allowed the identification and quantification of 13 cytostatics on four different regular surfaces: melamine-coated wood, phenolic compact, steel 304, and steel 316. This method proved adequate, allowing extremely low detection limits (down to 0.1 pg/cm^2^) and acceptable recoveries (average 80 + 12%, excluding CAP and DOX). Global uncertainty, necessary to correctly interpret results, was estimated to be below 35% for concentrations above 100 pg/cm^2^, except for CAP and DOX.

This method was successfully applied in a few areas from the preparation and administration wards of a university hospital located in northern Portugal. Although these preliminary results suggest contamination levels are generally low, the widespread presence of carcinogenic cytostatic CYC (the most detected, at concentrations up to 174 pg/cm^2^) evinces the necessity to review implemented procedures, alongside what has been commonly reported in other studies.

Furthermore, this study contributes to the future implementation of an environmental monitoring program as a useful tool to better protect workers through occupational exposure risk assessment.

## Figures and Tables

**Table 1:** Chromatographic and mass spectrometry information and validation parameters obtained for the instrumental analysis by liquid chromatography with tandem mass spectrometry (LC–MS/MS).

Cytostatic	rt (min)	Molecular Ion (*m/z*) (Cone Voltage, V)	Transition 1 (CE, eV)	Transition 2 (CE, eV)	Linearity (ng)	Regression Equation	R2	IDL ^1^ (pg/cm^2^)	IQL ^1^ (pg/cm^2^)	Accuracy ^2^ (Mean ± SD, %)	Precision (CV%) ^3^
BIC	37.802	429.00 [M − H]^−^ (−3.5)	429.00 → 255.05 (16)	429.00 → 184.95 (39)	0.2–20	Y = 0.270X + 0.001	0.9997	0.1	0.4	96 ± 6	7
CAP	36.451	360.20 [M + H]^+^ (4.5)	360.20 → 244.00 (−13)	360.20 → 174.00 (−23)	0.2–200	Y = 0.075X − 0.247	0.9976	0.3	1.0	251 ± 81	32
CYC	32.068	260.90 [M + H]^+^ (4.5)	260.90 → 139.95 (−23)	260.90 → 106.05 (−19)	0.2–200	Y = 0.012X + 0.000	0.9997	1.7	5.6	87 ± 4	5
CYPR	39.603	417.20 [M + H]^+^ (4.5)	417.20 → 357.15 (−18)	417.20 → 279.00 (−25)	1–200	Y = 0.003X − 0.001	0.9993	4.0	13.3	87 ± 10	11
DOX	34.483	544.00 [M + H]^+^ (4.5)	544.00 → 397.00 (−13)	544.00 → 361.00 (−28)	1–200	Y = 0.003X − 0.011	0.9982	0.9	3.1	24 ± 11	46
ETO	35.103	589.20 [M + H]^+^ (4.5)	589.20 → 228.95 (−20)	589.20 → 185.10 (−37)	0.2–100	Y = 0.005X − 0.001	0.9998	0.4	1.2	84 ± 10	11
FLU	38.796	275.00 [M − H]^−^ (−3.5)	275.00 → 201.95 (24)	275.00 → 205.05 (21)	0.2–20	Y = 0.059X + 0.019	0.9995	0.3	1.1	96 ± 14	14
IFO	30.343	260.90 [M + H]^+^ (4.5)	260.90 → 92.05 (−26)	260.90 → 153.95 (−23)	0.2–20	Y = 0.014X + 0.002	0.9998	1.6	5.3	82 ± 4	5
IMA	27.613	494.30 [M + H]^+^ (4.5)	494.30 → 394.05 (−27)	494.30 → 217.10 (−27)	0.2–200	Y = 0.050X + 0.010	0.9993	2.0 ^4^	6.7 ^4^	54 ± 14	25
MEG	39.874	385.10 [M + H]^+^ (4.5)	385.10 → 267.10 (−20)	385.10 → 325.15 (−15)	0.2–200	Y = 0.018X − 0.004	0.9998	0.6	1.9	83 ± 7	8
MMF	32.750	434.10 [M + H]^+^ (4.5)	434.10 → 114.05 (−27)	434.10 → 194.95 (−36)	0.2–200	Y = 0.131X − 0.021	0.9998	0.1	0.4	71 ± 7	10
PAC	39.091	876.20 [M + H]^+^ (4.5)	876.20 → 308.00 (−30)	876.20 → 591.15 (−28)	0.2–40	Y = 0.015X + 0.003	0.9987	0.4	1.2	68 ± 17	25
PRE	36.017	359.10 [M + H]^+^ (4.5)	359.10 → 146.95 (−26)	359.10 → 341.15 (−13)	1–200	Y = 0.004X − 0.002	0.9999	1.8	6.0	78 ± 6	8
CYC-d4	31.930	265.00 [M + H]^+^ (4.5)	265.00 → 140.00 (−24)	265.00 → 63.00 (−43)							

rt—retention time; CE—collision energy; IDL—instrumental detection limit; IQL—instrumental quantification limit; SD—standard deviation; CV—coefficient of variation; Y—ratio (area of cytostatic/area of internal standard); X—mass of cytostatic (ng); BIC—bicalutamide; CAP—capecitabine; CYC—cyclophosphamide; CYPR—cyproterone; DOX—doxorubicin; ETO—etoposide; FLU—flutamide; IFO—ifosfamide; IMA—imatinib; MEG—megestrol; MMF—mycophenolate mofetil; PAC—paclitaxel; PRE—prednisone; CYC-d4—cyclophosphamide-d4. ^1^ Mass (ng) values were divided by the standard sampling area (100 cm^2^). ^2^ Average of the recovery values obtained for the four surfaces studied. ^3^ CV from 16 measurements in different days and different samples. ^4^ Assumed the lowest calibration point as the IDL; IQL = 10/3 × IDL.

**Table 2:** Maximum and mean values for concentrations (pg/cm^2^) of the target cytostatics extracted from wipe samples of a preparation unit and an administration unit from a university hospital in northern Portugal.

Cytostatic.	Preparation Unit		Administration Unit	
	Maximum	Mean ± SD (Frequency)	Maximum	Mean ± SD (Frequency)
BIC	–	–	–	–
CAP	33	33 ± 0 (1/12)	–	–
CYC	174	60 ± 58 (10/12)	11	6 ± 3 (6/16)
CYPR	–	–	168	91 ± 55 (3/16)
DOX	–	–	–	–
ETO	–	–	37	37 ± 0 (1/16)
FLU	–	–	–	–
IFO	22	13 ± 7 (4/12)	4	4 ± 0 (1/16)
IMA	–	–	–	–
MEG	7	7 ± 0 (1/12)	5	4 ± 1 (5/16)
MMF	2	2 ± 0 (1/12)	3	3 ± 0 (1/16)
PAC	–	–	–	–
PRE	–	–	–	–

SD—standard deviation; BIC—bicalutamide; CAP—capecitabine; CYC—cyclophosphamide; CYPR—cyproterone; DOX—doxorubicin; ETO—etoposide; FLU—flutamide; IFO—ifosfamide; IMA—imatinib; MEG—megestrol; MMF—mycophenolate mofetil; PAC—paclitaxel; PRE—prednisone.

## Data Availability

Data is contained within the article.

## Appendix A. Estimation of the Global Uncertainty Associated with the Analytical Results

To evaluate the global uncertainty (*U*) associated with the quantification of cytostatics in surfaces by LC–MS/MS, the bottom-up approach proposed by the International Organization for Standardization and adopted by the EURACHEM-CITAC Guide was applied [31]. Global uncertainty is believed to be mainly affected by four sources of uncertainty and is expressed as

(A1) $$U=\sqrt{U1^{2}+U2^{2}+U3^{2}+U4^{2}},$$

where

1. *U*1 is the uncertainty associated with the preparation of standards (estimated using the error propagation law for the different dilution steps from the stock standard solution),

(A2) $$U1=\sqrt{\sum_{i}\left(\frac{\Delta mi}{mi}\right)}.$$

Δ*mi*—error associated with the measurement equipment; *mi*—measured value.

2. *U*2 is the uncertainty associated with the calibration curve (calculated for the different mass levels of the standards),

(A3) $$U2=\frac{sx_{0}}{x_{0}}=\frac{1}{x_{0}}\frac{s_{y/x}}{a}\sqrt{\frac{1}{m}+\frac{1}{n}+\frac{{\left(y_{0}-\bar{y}\right)}^{2}}{a^{2}\sum_{i}{\left(x_{i\;}-\bar{x}\right)}^{2}}}=\frac{1}{x_{0}}\frac{1}{a}\sqrt{\frac{\sum_{i}{\left(y_{i\;}-y_{0}\right)}^{2}}{n-2}}\sqrt{\frac{1}{m}+\frac{1}{n}+\frac{{\left(y_{0\;}-\bar{y}\right)}^{2}}{a^{2}\sum_{i}{\left(x_{i\;}-\;\bar{x}\right)}^{2}}}.$$

*sx*_0_—standard deviation of the concentration; *x*_0_—mass; *a*—slope of the calibration curve (*y* = *ax* + *b*); *m*—number of replicates performed; *n*—number of standards to build the calibration curve; *y*_0_—*y* values calculated by the calibration curve from *x* values; $\bar{y}$—average of *y_i_* (experimental) values of the calibration curve standards; *x_i_*—concentration of each standard used in the calibration curve; $\bar{x}$—average of *x_i_* values; *y_i_*—experimental values of the calibration curve standards.

3. *U*3 is the uncertainty associated with the precision of the method (estimated as the average result of the relative standard deviation of recovery),

(A4) $$U3=\frac{s}{\bar{y}\sqrt{n}}$$

*s*—standard deviation of experimental *y* values in precision assays; $\bar{y}$—average of experimental *y* values in precision assays; *n*—number of precision assays.

4. *U*4 is the uncertainty associated with the accuracy (calculated as the average percent recovery obtained within all the experiments),

(A5) $$U4=\frac{s\left(\eta \right)}{\sqrt{n}}$$

*sη*—relative standard deviation of the average percent recovery; *n*—number of accuracy assays.
